# Machine learning algorithm performance evaluation in structural magnetic resonance imaging-based classification of pediatric bipolar disorders type I patients

**DOI:** 10.3389/fncom.2022.915477

**Published:** 2022-08-23

**Authors:** Ruhai Dou, Weijia Gao, Qingmin Meng, Xiaotong Zhang, Weifang Cao, Liangfeng Kuang, Jinpeng Niu, Yongxin Guo, Dong Cui, Qing Jiao, Jianfeng Qiu, Linyan Su, Guangming Lu

**Affiliations:** ^1^Department of Radiology, Shandong First Medical University and Shandong Academy of Medical Sciences, Taian, China; ^2^Department of Child Psychology, The Children’s Hospital, Zhejiang University School of Medicine, Hangzhou, China; ^3^Department of Interventional Radiology, Taian Central Hospital, Taian, China; ^4^Institute of Biomedical Engineering, Chinese Academy of Medical Sciences and Peking Union Medical College, Tianjin, China; ^5^Key Laboratory of Psychiatry and Mental Health of Hunan Province, Mental Health Institute of the Second Xiangya Hospital, Central South University, Changsha, China; ^6^Department of Medical Imaging, Jinling Hospital, Clinical School of Medical College, Nanjing University, Nanjing, China

**Keywords:** pediatric bipolar disorder, machine learning, logistic regression, support vector machine, random forest, naïve Bayes, k-nearest neighbor, adaptive boosting classifier

## Abstract

The diagnosis based on clinical assessment of pediatric bipolar disorder (PBD) may sometimes lead to misdiagnosis in clinical practice. For the past several years, machine learning (ML) methods were introduced for the classification of bipolar disorder (BD), which were helpful in the diagnosis of BD. In this study, brain cortical thickness and subcortical volume of 33 PBD-I patients and 19 age-sex matched healthy controls (HCs) were extracted from the magnetic resonance imaging (MRI) data and set as features for classification. The dimensionality reduced feature subset, which was filtered by Lasso or f_classif, was sent to the six classifiers (logistic regression (LR), support vector machine (SVM), random forest classifier, naïve Bayes, k-nearest neighbor, and AdaBoost algorithm), and the classifiers were trained and tested. Among all the classifiers, the top two classifiers with the highest accuracy were LR (84.19%) and SVM (82.80%). Feature selection was performed in the six algorithms to obtain the most important variables including the right middle temporal gyrus and bilateral pallidum, which is consistent with structural and functional anomalous changes in these brain regions in PBD patients. These findings take the computer-aided diagnosis of BD a step forward.

## Introduction

Bipolar disorder (BD) is a common category of mental disorder, which is featured as a cycling pattern of mood states with manic or hypomanic episodes, depressive episodes, and euthymia. BD affects about 2% of adolescents under the age of 18 (Frias et al., 2015). Recurrent pediatric bipolar disorder (PBD) has a significant impact on patients’ daily life. Under the current diagnostic manual, PBD can be classified as manic (PBD-I) or hypomanic (PBD-II), based on the absence of a complete manic episode. Currently, in clinical practice, there are still great challenges in the management of BD, the diagnosis of which is solely based on clinical evaluation, and it may lead to a misdiagnosis that the diagnosis result is affected by many factors, such as the subjectivity of symptoms and the description of patients. In recent years, machine learning (ML) methods have been introduced into the classification of BD, which have proved helpful for the diagnosis of BD.

For distinguishing BD patients from normal subjects, commonly used ML methods include logistic regression (LR) (Pirooznia et al., 2012), support vector machine (SVM) (Schnack et al., 2014), random forest classifier (RF) (Besga et al., 2015; Chuang and Kuo, 2017), naïve Bayes (NB) (Struyf et al., 2008), k-nearest neighbor (kNN) (Struyf et al., 2008; Acikel et al., 2016), and so on. Currently, there are relatively few studies on using structural magnetic resonance imaging (sMRI) for BD discrimination, which mainly focused on gray matter (GM) and white matter (WM) density as features to train classifiers with different accuracy rates. In research using GM and WM to train the relevance vector machine (RVM) algorithm to distinguish BD from healthy controls (HCs), the accuracy of RVM for only using density data of WM, GM, and a combination of WM and GM was 70.3, 64.9, and 64%, respectively (Cao et al., 2018). When Gaussian process classifier (GPC) was trained by GM and WM data from sMRI of cortical and subcortical structures to distinguish BD type from HCs, the accuracy of 73% in cohort 1 and 72% in cohort 2 for GM, and accuracy of 69% in cohort 1 and 78% in cohort 2 for WM were obtained (Rocha-Rego et al., 2014). Doan et al. (2017) trained an RF classifier to distinguish BD from HCs with an accuracy of 66%, using a data-driven fusion of cortical thickness, density maps of GM, and surface area as features. Using morphometric features of the voxel-based GM in the bilateral amygdala, Mwangi et al. (2014) trained the ElasticNet algorithm to distinguish BD from HCs with an accuracy rate of 78.12%. A wide range of commonly used ML algorithms was trained to differentiate individuals with BD from HCs using GM voxel-based morphometry, achieving an accuracy of 62.3% for Ridge, 65.5% for Lasso, 63.5% for ElasticNet, 65.1% for L0-norm regularization, 64.7% for SVM, 61.6% for regularized discriminant analysis, 60.8% for GPC, and 62% for RF (Salvador et al., 2017).

Support vector machine is most frequently used among many ML methods for distinguishing BD from HCs. For example, Nunes et al. (2020) employed brain regional cortical thickness, surface area, and subcortical volumes to train the linear kernel SVM algorithm to delineate BD from HCs and obtain an accuracy of 58.67%. Other studies reported accuracy of 60 and 66.1% when SVM was trained by GM density and volume of GM and WM in distinguishing BD from HCs (Schnack et al., 2014; Serpa et al., 2014). When the regional mean GM volume of 14 clusters and two brain regions (right anterior cingulate cortices and left inferior frontal gyrus) were utilized as features to input into the SVM classifier to distinguish between BD and HCs, the accuracy was improved to 89.03–90.69% (Lin et al., 2018) and 88.1% (Matsuo et al., 2019). As can be seen from the above description, the current identification research of BD using sMRI mainly focuses on SVM, and the performance comparison of other various classifiers is worse than that of SVM.

Consequently, the goal of this study is to evaluate whether several classical ML algorithms could effectively classify PBD-I patients and HCs from sMRI, and of these, the ML algorithm performed best on these data and analyzed the contribution of different brain regions to classification. Different feature selection methods were applied for different ML methodologies. The parameters of different methodologies were reported, the most important structural indices of brain regions were identified, and the performance comparison among the above algorithms was discussed.

## Materials and methods

### Participants

In this study, 33 manic PBD-I patients and 19 HCs were included. All BD patients were out-patients from the Clinical Psychiatric Department in the Second Xiangya Hospital of Central South University, and HCs were recruited by advertisements in local schools. All the BD patients met the criteria of the Diagnostic and Statistical Manual of Mental Disorders 4th Edition (DSM-IV). The inclusion criteria of all subjects were as follows: (a) right-handed; (b) 12–18 years old; (c) should be able to keep their head still to complete the MRI scan. The exclusion criteria for all participants were as follows: (a) full-scale intelligence quotient (IQ)’ score ≤80; (b) must be pregnant; (c) contraindications to MRI scan, including foreign metal in the body and claustrophobia; (d) history of drug abuse and alcoholism; (e) electroconvulsive therapy history; (f) other mental diseases, including learning disorder, nervosa anorexia or bulimia, split personality, and autism; (g) active medical or neurological malady. In addition, HCs were required to have no history of mental disorders among their first-degree relatives. The Stroop color-word test (SCWT) was performed on all subjects before MRI scanning. SCWT measures suppress habitual response patterns, working memory, and selective attention. The test consists of three parts (SCWT-1, SCWT-2, and SCWT-3), each part involves 100 visual stimuli. Detailed information about this test may be found in other literature (Kuang et al., 2020). The data were collected from January 2012 to July 2014.

This research was approved by the Ethics Committee of the Second Xiangya Hospital of Central South University. Informed consent papers were obtained from all participants and one statutory guardian at least. The difference in sex distribution between groups was evaluated with a chi-square test, whereas the differences in the mean of age between PBD and HC groups were evaluated using unpaired two-sample *T*-tests. Statistical analysis was performed with python 3.7.8 (SPSS25 for windows).

### Magnetic resonance imaging acquisition

#### Magnetic resonance imaging scan

In this study, the Siemens 3.0 T scanner (Siemens, Munich, Germany) was used for MRI data acquisition. During the MRI scan, the subjects were asked to stay on their backs and still, close their eyes, and not think or fall asleep. To protect the subject’s hearing from the noise of the scanner, each subject was equipped with cotton earplugs. Using a three-dimensional magnetization-prepared rapid gradient-echo sequence, a T_1_-weighted image of the entire head was obtained. The scan parameters were set as follows: repetition time (TR) = 2,300 ms, echo time (TE) = 2.03 ms, inversion time (TI) = 900 ms, thickness = 1 mm, gap = 0 mm, field of view (FOV) = 256 mm × 256 mm, flip angle = 9°, and matrix = 256 × 256.

### Magnetic resonance imaging pre-processing and volume and cortical thickness calculation

Subjects were excluded through visual inspection of the 3D-T1 weighted images: failure of FreeSurfer pre-processing and aliasing artifact generated by a head motion. After quality control, 33 out of 36 PBD-I patients, and 19 HCs were included in our study. To check differences in image quality and head motion between cases and controls, the Euler number was calculated in each T1w image, and to quantitatively assess the image quality, a two-sample *T*-test was used (two-sided) (Rosen et al., 2018). There was no significant difference in the Euler number between the two groups (*T*-value = 0.039, *P*-value = 0.969) (Supplementary Figure 1).

We pre-processed all T_1_-weighted images using the FreeSurfer software (v6.0).^1^ In the FreeSurfer, we conducted the main recon stream (“recon-all”) for the calculation of cortical thickness, subcortical volume, and total intracranial volume (TIV). This process included the following steps: (a) Motion correction was performed to minimize the impact of head movement during the scan; (b) The skull was stripped and the brain was extracted; (c) To implement the affine transformation of the original volume to MNI305 atlas, Talairach transformation was performed; (d) Intensity normalization was carried out to decrease the intensity difference caused by an inhomogeneous magnetic field or other factors in the same tissue; (e) T_1_-weighted images were divided into GM, WM, and cerebrospinal fluid (CSF); and (f) Transformation was conducted by linear transformation array format. Moreover, boundary subdivisions were made between GM and WM. For guaranteeing appropriate division of the cortical regions and subcortical structures, a trained doctor re-examined all subdivided boundaries and manually correct them if necessary. Then the volumes of subcortical structures, cortical GM thickness, and estimated TIV (eTIV) of each subject were calculated.

### Machine learning analysis

**FIGURE 1:**
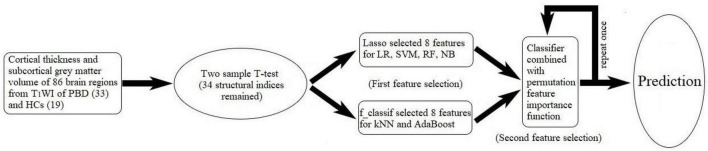
Workflow of machine learning in our work. In machine learning, the six classifiers are as follows: logistic regression (LR), support vector machine (SVM), random forest classifier (RF), naïve Bayes (NB), k-nearest neighbor (kNN), and AdaBoost algorithm (AdaBoost).

Machine learning technology is becoming more and more popular because of its powerful modeling ability. The ML classification task in this study is a process of supervised learning, the typical workflow is as follows (Figure 1): First, by processing the T_1_WI data in the FreeSurfer, cortical thickness and subcortical GM volume of 86 brain regions were set as features of ML classification. Second, the feature dimensionality is reduced by extracting meaningful features. An excellent feature subset can make it easier to build a predictive model, and the created model is also easier to complete the required tasks. And a poor feature subset requires a more complex model to achieve the same performance. Third, the dimensionality-reduced feature subset is randomly divided into training data and test data. The training data with class labels are sent to a classification algorithm (classifier), and the classifier uses the input data to create a model that can assign the right labels to observations. For evaluating the accuracy of the classifier, test data are provided to the classifier to evaluate its performance. In this study, using the scikit-learn package (0.24.1) in Python (v3.7.8), we developed six different machine algorithms: LR, SVM, RF, NB, kNN, and AdaBoost.

#### Logistic regression

Logistic regression is popular for its simplicity, parallelization, and interpretation. In the LR model, a logistic function is used for modeling. Regularization is a general algorithm and idea, so any algorithm that can produce overfitting may use regularization to avoid overfitting, and the same is true for LR with optional *l*_1_ regularization, *l*_2_ regularization, or elastic-net regularization. In this study, LR was built with *l*_2_ regularization and the inverse of the regularization coefficient was set as 1 (C = 1).

#### Support vector machine

Support vector machine (Cortes and Vapnik, 1995) is a generalized linear classifier in the supervised learning method. In a high- or infinite-dimensional space, SVM constructs a hyper-plane or a set of hyper-planes for classification and regression. Its decision boundary is the maximal margin hyperplane of the training data. The decision function of SVM depends on a certain subset of training data, which is called a support vector.

Support vector machine has been applied in various fields, such as text classification, bioinformatics, image processing, cancer identification, handwritten character recognition, and so on. SVM has many advantages including: (1) it is a learning method for small samples with a solid theoretical foundation; (2) the final decision function of SVM is only determined by a few support vectors, and the complexity of calculation depends on the number of support vectors rather than the dimension of the sample space, which avoids the curse of dimensionality in a sense, so it can work in high dimensional spaces; (3) it still works even if the dimension is larger than the sample size; (4) a small number of support vectors are insensitive to outliers, which can not only help us grasp key samples and remove a large number of redundant samples but also ensure that method is simple in algorithm and has good “robustness.”

#### Random forest

Random forest (Breiman, 2001) is composed of multiple decision tree classifiers that are used to train and predict samples. In RF, each tree is built from a sample drawn with replacement from the training data, and the average is used to improve prediction accuracy and control overfitting. The advantages of RF are as follows: (1) the learning process is fast, and it is still effective on large databases; (2) it is not sensitive to multicollinearity; (3) the results are relatively robust for missing data and unbalanced data; (4) it can also be predicted accurately when there are many characteristic variables without variable selection; (5) it can evaluate the importance of features in the classification. Due to the above advantages, RF has been widely used in various fields, such as bioinformatics, ecology, medicine, proteomics, finance, and so on.

In this study, RF was made up of 100 trees in the forest. During the construction of a tree to split each node, the value of the number of variables is equal to the square root of the features number for the best split.

#### Naïve Bayes

Naïve Bayes classifier (Domingos and Pazzani, 1997) is one of the most widely used classifiers. A NB classifier is a supervised learning algorithm based on the Bayes theorem with attribute conditional independence assumption. In other words, it assumed that each attribute independently affects the classification results. Based on the Bayes theorem, the relationship between class variable *y* and dependent feature vector x (x_1_, ⋯, x_*n*_) can be obtained:


$$P\,\left(y|\text{x}\right)=\frac{P\,\left(y\right)\,P\,\left(\text{x}|y\right)}{P\,\left(x\right)}$$

Based on attribute conditional independence assumption


$$P\,\left(x_{i}|y,x_{1},\cdots ,x_{i-1},x_{i+1},\cdots ,x_{n}\right)=P\,\left(x_{i}|y\right)$$

the relationship between *y* and *x* can be rewritten as


$$P\,\left(y|\text{x}\right)=\frac{P\,\left(y\right)}{P\,\left(x\right)}\,\prod_{i=1}^{n}P\,\left(x_{i}|y\right)$$

The main difference among different Naive Bayes classifiers is the assumption distribution of *P*(*x*_*i*_|*y*). Whereas the assumptions of NB classifiers are obviously oversimplified, they work well in many real-world situations, such as patient classification, account classification, and so on.

In this study, we adopt a Gaussian NB classifier and the feature distribution*P*(*x*_*i*_|*y*) is assumed to be Gaussian as shown below.


$$P\,\left(x_{i}|y\right)=\frac{1}{\sqrt{2\,\pi \,\sigma_{y}^{2}}}\,\exp\left(-\frac{{\left(x_{i}-\mu_{y}\right)}^{2}}{2\,\sigma_{y}^{2}}\right)$$

#### K-nearest neighbor

The theory of the kNN algorithm (Peterson, 2009) is relatively mature, and it is also a commonly used supervised learning algorithm. It does not try to build a general internal model, but only stores training data. A test sample was given based on a certain distance metric (such as Euclidean distance), and the nearest k-training samples to the test sample were found in the training set. Then the classification of the test sample was predicted according to the information of these k “neighbors.” Generally, the “voting method” is utilized in the classification, the category label that appears most in the k sample is selected as the prediction result. In the kNN classifier, *k* is an important parameter. When *k* takes different values, the classification results will be significantly different. The optimal choice of the value *k* is selected by cross-validation. In general, a larger *k* can obtain a higher signal-to-noise ratio (Lemm et al., 2011) but the definition of the classification boundary was reduced.

#### Adaptive boosting classifier

AdaBoost algorithm (Freund and Schapire, 1997) is a typical iterative learning algorithm, whose core idea is to train a series of weak classifiers for the same training set, and then collect these weak classifiers to construct a stronger final classifier. The AdaBoost algorithm has been proven to be an effective and practical boosting algorithm. The process of the AdaBoost algorithm is as follows: Initially, a first weak classifier is obtained by learning *N* training samples (the original data), and all the samples have the same weight 1/*N*. Second, the misclassified sample weight of the first weak classifier is increased, whereas the weights of the samples that were predicted correctly are decreased. The re-weighed *N* training samples are used to train the second weak classifier. For each training, the weight of the sample is separately modified, and the weak classifier is trained by the re-weighted *N* training samples. This process is repeated until the number of the weak classifier reaches a pre-specified value. Finally, a strong classifier is obtained by combining all the weak classifiers obtained from each training and the predictions from all classifiers combined to produce the final prediction. In the prediction, the weight of the weak classifier with a small classification error rate is increased, which plays a larger role in the strong classifier, whereas the weight of the weak classifier plays a smaller role with a large classification error rate is decreased.

### Statistical feature extraction

For each subject in this study, a total of 87 brain structural indices were obtained through the image processing steps described previously. Among them, eTIV was linearly regressed as a covariant, and structural indices of 86 brain regions (20 subcortical volumes and 66 cortical GM thickness) were retained. A two-sample *T*-test was conducted on structural indices of 86 brain regions between the BD-I and HCs groups. Before the two-sample *T*-test, the equality of variance test was performed by the Levene method. If equality of variance was satisfied, the two-sample *T*-test was carried out with parameter equal_var (a function *T*-test_ind for the two-sample *T*-test) to be true, otherwise, equal_var was set to be false. With eTIV as a regressor, the dimensionality of the structural indices dataset was reduced by the *T*-test, and 34 structural indices remained.

### Feature selection

Variable and feature selection are the focus of research in many application fields, where datasets may have thousands of variables and features (Zheng and Wang, 2017). The benefits of variable and feature selection are 4-fold: reducing the number and dimension of features, reducing the difficulty of learning tasks, reducing training time, and improving the prediction performance of the predictor (Guyon and Elisseeff, 2003).

In classification, the curse of dimensionality is more severe if the number of samples is less than the number of features. Therefore, feature selection must be carried out to reduce feature dimension and avoid the curse of dimensionality. For building predictors, selecting the most relevant variables is usually not the best choice, especially when the variables are redundant. On the contrary, the subset of useful variables is often related variables after excluding many redundant ones (Baggenstoss, 2004). Under the premise of not significantly reducing the classification accuracy, the feature subset obtained should be as small as possible, which may ensure stability and strong adaptability.

To overcome over-fitting, eliminate redundant items, and achieve the best prediction performance, we applied different methods to select features. Lasso (Tibshirani, 2011) was used to select features for the LR, SVM, RF, and NB, and to assign weights to features (the coefficients of a linear model) in this study. Lasso is a linear model with the *l*_1_ regularization to estimate sparse coefficient, and is very useful because it tends to choose solutions with fewer non-zero coefficients, hence it can effectively reduce the number of features.

In python, the function f_classif was used to compute the ANOVA *F*-value for the provided sample. The function f_classif is placed in the function SelectkBest in scikit-learn as a scoring function, which removes all features except the *k* features with the highest score. Features selected by f_calssif were used for kNN and AdaBoost. The important value of each feature was calculated using the permutation feature important function in Python. After the second feature selection, each classifier of the five classifiers was rebuilt only on the most important features. In this study, permutation testing of the feature selection (5,000 times) was conducted to ensure the robustness of the features.

### Cross-validated accuracy

To assess both feature selection results and classification performance, a *k*-fold cross-validation repeated *p* times (repeated *K*-fold in scikit-learn) method may be used for evaluating and comparing learning algorithms. In the process of *k*-fold cross-validation repeated *p* times, all the data are divided randomly into *k* groups of data. When using *k*−1-folds trains the model, and the data left is used for verifying the model, the process is repeated for p times. In addition, the method of k-fold cross-validation repeated *p* times may be useful in comparing the performance of different ML algorithms on the same dataset for the purpose of selecting a better algorithm for the data under consideration.

In this study, a 2-fold cross-validation repeated four times was used, splitting the dataset into a training set of 50% and a test set of 50%. The process was repeated four times and the performance of different classification algorithms was compared.

## Results

### Clinical and magnetic resonance imaging findings

Demographic and clinical characteristics of PBD-I patients and HCs are shown in Table 1. No significant difference was found in gender, age, and education years between the two groups. Significant differences were found in the scores of SCWT.

**TABLE 1:** Demographic and clinical characteristics PBD1 and HC groups.

Characteristics	PBD-I (*n* = 33)	Healthy controls (*n* = 19)	*T/*χ^2^	*P*-value
Gender (M/F)	18/15	9/10	1.51^#^	0.21^#^
Age (years)	15.12 ± 1.84	14.15 ± 1.57	1.90^∧^	0.06^∧^
Education (years)	8.30 ± 1.91	7.47 ± 2.22	1.42^∧^	0.16^∧^
Onset age (years)	13.69 ± 1.82	–		
Illness duration (months)	18.69 ± 13.43	–		
Number of episodes	3.57 ± 2.31	–		
SCWT1	52 ± 15.12	66 ± 12.25		0.001^∧^
SCWT2	67 ± 18.66	88 ± 9.07		<0.001^∧^
SCWT3	31 ± 8.04	41 ± 9.42		<0.001^∧^

Data were shown in mean ± standard deviation.

^#^Pearson chi-square test.

^∧^Two-sample *T*-test.

Totally, structural indices of 86 brain regions, including volumes of 20 subcortical structures and GM thicknesses of 66 cortical regions were obtained. With the eTIV as a regressor, a *T*-test was conducted on the 86 structural indices (86 features) for evaluating brain regions showing significant differences between PBD-I patients and HCs. As a result, 34 features were saved after the *T*-test and would be used for further research.

### Feature selection

All of the 34 features were input into Lasso that tends to select solutions with fewer non-zero coefficients, which effectively reduces the number of features relied on by the given solution; eight features (Feature group A) with non-zero coefficients were selected including the right middle temporal gyrus (MTG.R), right Pallidum (Pallidum.R), left Pallidum (Pallidum.L), right amygdala (AMG.R), right transverse temporal gyrus (TTG.R), left transverse temporal gyrus (TTG.L), left lateral occipital gyrus (LOG.L), and right postcentral gyrus (PosCG.R). The eight features of Feature group A selected by Lasso were used to train the models LR, SVM, RF, and NB.

**FIGURE 2:**
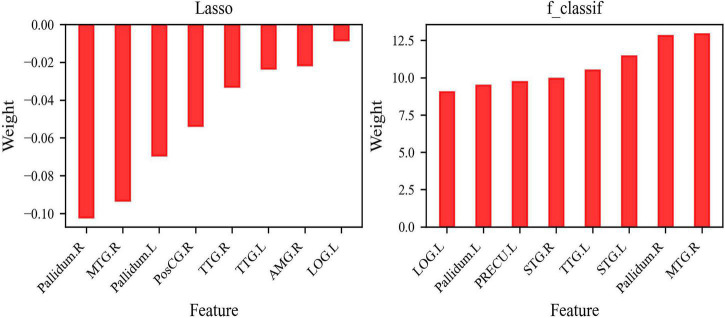
Features selection. **(A)** Eight features selected by Lasso; **(B)** eight features selected by f_classif.

According to the result of f_classif, which was conducted to calculate the ANOVA *F*-value between the label and each feature (34 features in total), the other eight features [Feature group B: MTG.R, Pallidum.R, Pallidum.L, right superior temporal gyrus (STG.R), left STG (STG.L), TTG.L, LOG.L, left precuneus (PRECU.L)], with the highest ANOVA *F*-value were selected. The larger the ANOVA *F*-value of the feature was, the smaller the *P*-value of the feature would be, and the stronger the prediction ability of the feature would be. kNN and AdaBoost were trained by the features of Feature group B. Figure 2 describes the weight of Feature group A in Lasso and Feature group B in f_classif.

Table 2 demonstrates the classification parameters (accuracy, sensibility, specificity, and AUC) from the step of 2-fold cross-validation repeated four times when using the cortical thickness or volume of the 11 brain regions in feature group A and feature group B for classification. The values in Table 2 are the average of the parameters in all of the 2-fold cross-validation repeated four times. The accuracies of LR and SVM were above 80% (LR: 82.24% and SVM: 80.08%), and that of RF, NB, kNN, and AdaBoost were above 75% (RF: 79.53, NB: 78.72%, kNN: 77.74%, and AD: 77.66%). AdaBoost had the lowest accuracy, whereas LR had the highest accuracy.

**TABLE 2:** Two-fold cross-validation repeated four-time accuracy, sensibility, specificity, and AUC calculated for each classifier with different features.

Algorithm	Features*	Accuracies (%)	Sensibility (%)	Specificity (%)	AUC
LR	A	82.24	89.26	70.11	0.79
	B	84.19	93.91	67.32	0.80
	C	85.41	90.85	76.89	0.83
SVM	A	80.08	83.49	73.57	0.78
	B	82.80	91.20	68.13	0.79
	C	84.57	87.89	77.29	0.82
RF	A	79.53	90.90	57.94	0.74
	B	84.59	91.13	70.27	0.81
	C	81.86	89.17	68.61	0.78
NB	A	78.72	90.40	58.20	0.74
	B	83.56	91.20	71.01	0.80
	C	83.64	90.29	71.76	0.81
kNN	A	77.74	91.66	55.04	0.73
	B	78.70	89.84	59.64	0.74
	C	81.86	88.92	68.47	0.78
AdaBoost	A	77.66	85.79	64.10	0.74
	B	78.24	84.35	67.50	0.75
	C	77.33	84.96	64.81	0.74

Classification indices obtained with eight features, six features, combined six features, and Stroop color-word test scores were represented by the percentage values in the table.

*A: Classification using eight features, including cortical thickness of MTG.R, LOG.L, PosCG.R, bilateral TTG, and gray matter volume of AMG.R and bilateral pallidum.

B: Classification using six features, including cortical thickness of MTG.R, bilateral TTG, and gray matter volume of AMG.R and bilateral pallidum.

C: Classification using features combining the structural MRI indices of the six brain regions and SCWT scores.

### Feature importance evaluation

**FIGURE 3:**
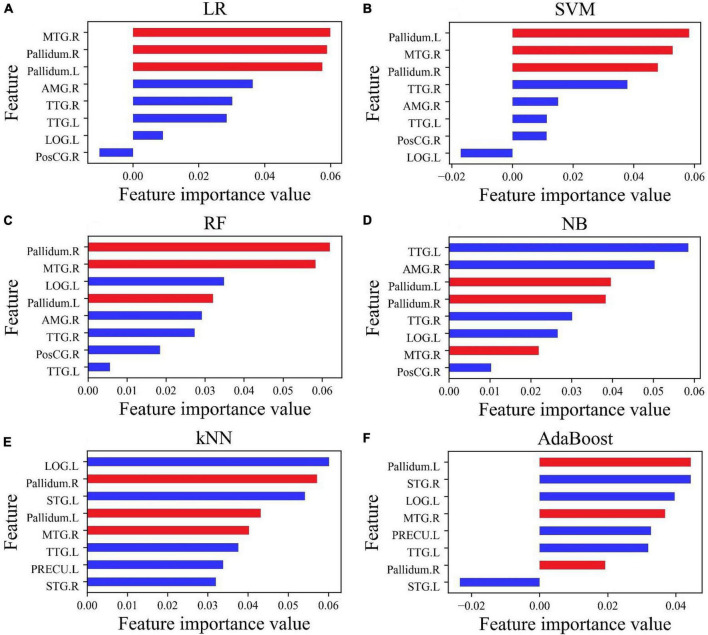
The importance value of features of eight features selected by Lasso **(A–D)** and f_classif **(E,F)**. The three most important features (MTG.R, Pallidum.R, and Pallidum.L) affecting the accuracy of classification have been marked in red.

For improving the accuracy of prediction, selecting few features, and losing as little information as possible, the permutation feature importance function in Python was used to evaluate the importance of each feature in each classifier. The result is shown in Figure 3. The feature importance in the six classifiers had a hierarchical distribution. In LR and SVM, brain regions showing the top three feature importance values were Pallidum.L, Pallidum.R, and MTG.R (highlighted in red), followed by TTG.L, TTG.R, and AMG.R, and with the lowest level in the LOG.L and PosCG.R (Figures 3A,B). It may be seen that the feature importance value of PosCG.R in Figure 3A and that of LOG.L in Figure 3B are less than 0, which may reduce the accuracy of LR and SVM.

In different classifiers, the feature importance value ranking of each brain area is different. In RF, the feature importance value ranking is MTG.R, Pallidum.R, LOG.L, Pallidum.L, AMG.R, TTG.R, PosCG.R, and TTG.L (Figure 3C). In NB, the ranking is TTG.L, AMG.R, Pallidum.L, Pallidum.R, TTG.R, LOG.L, PosCG.R, and MTG.R (Figure 3D). In kNN, the first three feature importance values are in LOG.L, Pallidum.R, and STG.L, followed by Pallidum.L, MTG.R, and TTG.L, and with the lowest level in PRFCU.L and STG.R (Figure 3E). In AdaBoost, the first three feature importance values are in Pallidum.L, STG.R, and LOG.L, followed by MTG.R, PRFCU.L, and TTG.L, and with the lowest level in Pallidm.R and STG.L (Figure 3F). It may be seen that the feature importance value of STG.L in Figure 3F is less than 0, which may reduce the accuracy of AdaBoost.

### Machine learning algorithms on six features

The curse of dimensionality has always been an issue in the ML research (Quintero et al., 2021; Zhang et al., 2021). For classification, there are not enough data objects to create a model to reliably assign all possible objects to a class. As a result, for high-dimensional data with low sample space, the accuracy of classification is reduced. In this study, the feature importance values of PosCG.R and LOG.L are less than zero, which reduces the accuracy of classifications. To obtain an even more accurate classification, we eliminated features LOG.L and PosCG.R, and only retained the remaining six features selected by Lasso. Permutation testing showed the robustness of the six features (*P* < 0.05, FDR corrected).

Four classifiers (LG, SVM, RF, and NB) were used to reclassify the feature dataset filtered by Lasso. The accuracies of the four classification algorithms had been improved: LR from 82.28 to 84.19%, SVM from 80.08 to 82.8%, RF from 79.53 to 84.59%, and NB from 78.72 to 83.56%. In the feature dataset selected by f_classif, we removed two features STG.L and PRECU.L. The feature importance value of STG.L in Figure 3F is less than 0, which will reduce the accuracy of classifications. And for both kNN and AdaBoost classifiers, the feature importance value of PRECU.L (Figures 3E,F) ranks relatively low and contributes little to classifications. The accuracy of the other two classifiers had been also improved: kNN from 77.74 to 78.70% and AdaBoost from 77.66 to 78.24% (Table 2). The accuracies of all classifiers had been slightly improved; the accuracies of LR, SVM, RF, and NB are more than 80%, but the accuracies of kNN and AdaBoost were less than 80%.

**FIGURE 4:**
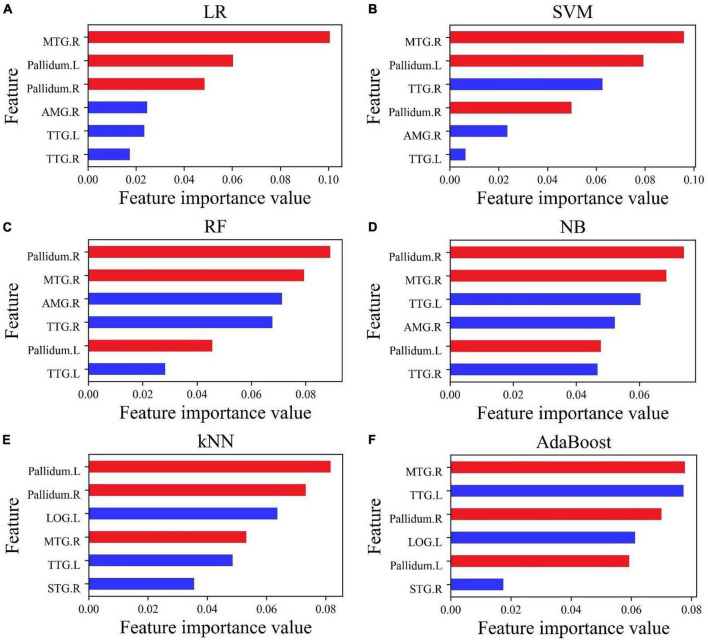
The importance of feature ranking for six features. **(A–D)** Six features selected by Lasso; **(E,F)** six features selected by f_classif. The three features MTG.R, Pallidum.R, and Pallidum.L marked in red have important effects on the accuracy of the classification.

Although the accuracy of all classifiers had been improved, the ranking of the importance of features had also changed, and the importance value of all features was greater than zero, as shown in Figure 4. The importance of features MTG.R, Pallidum.R, and Pallidum.L (marked in red) became more prominent. The top three features in all the six classifiers included at least two of the above three features.

Combining SCWT scores and MRI data, including the cortical thickness of MTG.R and bilateral TTG, GM volume of AMG.R and bilateral pallidum, as the classifying feature, the six classifiers were retrained and achieved better accuracy than those using MRI data alone (Table 2).

## Discussion

In this study, a variety of classifiers (LR, SVM, RF, NB, kNN, and AdaBoost algorithm) were applied to the cortical thickness and substructural volume extracted from the T_1_ MRI image, with the purpose to evaluate how well they performed in discriminating between PBD-I patients and HCs, which may help for the early diagnosis of PBD-I. The features selected by Lasso and f_classif were used to train the six classifiers, and prediction accuracies of more than 75% were obtained. After filtering according to the permutation feature importance function, six features were left and utilized to retrain the six classifiers, and the accuracy of the classifiers was greatly improved. The accuracy of kNN and AdaBoost was more than 78%, and that of LR, SVM, RF, and NB was more than 80%. The accuracy of RF was the highest, reaching 84.59%.

For the distinction between PBD-I and HCs, a total of six classifiers, namely LR, SVM, RF, NB, kNN, and AdaBoost, were trained by eight features in the process of *k*-fold cross-validation repeated *p* times (see Table 2). The performances of classifiers were very different because they came from different algorithm methods. The method of LR performed the best with a high accuracy (82.24%), which suggests the features we screened were more friendly to the linear classifier. The accuracies of SVM and RF were similar (80.08 and 79.53%). SVM was the most popular ML algorithm in practice and was suitable for small samples, which does not mean that the absolute number of samples was small, but the number of samples required by the SVM algorithm was relatively small as compared to the complexity of the problem. Whereas RF was still valid on large databases, and for missing data and unbalanced data, the results were relatively robust. In our work, the accuracy of SVM and RF were almost the same, which was due to using a *k*-fold cross-validation repeated *p* times. In addition, we used Gaussian NB, which is inherently weaker than other classifiers, such as SVM, RF, and LG. Its accuracy was not very ideal, because it assumes that the prior probability of the feature vector is Gaussian distribution. In our work, the unequal number of BD and HCs has some adverse effects on the accuracy of kNN (77.74%). The accuracy of AdaBoost was the worst (77.66%). To achieve higher prediction accuracy, the AdaBoost algorithm needs a large training sample set, on which the *k*-fold cross-validation repeated *p* times had limited improvement.

Features selected by Lasso were utilized for the classifiers LR, SVM, RF, and NB (Figure 2A) and those selected by f_classif were used in training kNN and AdaBoost (Figure 2B). There were five shared features in the two groups of features: MTG.R, Pallidum.R, Pallidum.L, TTG.L, and LOG.L. For classifiers LR, SVM, NB, RF, kNN, and AdaBoost, the first three important features contain one or two or even all the three features: MTG.R, Pallidum.R, and Pallidum.L (shown with red bars in Figure 3), which was roughly consistent with the order of importance of the three brain regions shown in Figure 2. In addition, for improving the accuracy of the classifiers, we applied feature selection to obtain the weight value of the features, so the features were screened twice. The value of feature importance was calculated by the permutation feature importance function. The importance values of two features, LOG.L, and PosCG.R, were less than 0, which would affect the accuracy of the classifiers. Thus, the two features were removed in classifiers LR, SVM, NB, and RF, and the feature subset was reduced from eight features to six features. For the classifier kNN and AdaBoost, we removed STG.L and PRECU.L, whose importance value was less than 0 or small. Six classifiers retrained by a new feature subset including six features obtained very excellent results, the accuracy of kNN (78.70%) and AdaBoost (78.24%) was improved by 1%, and the accuracy of the other four classifiers exceeded 80%, as shown with data of feature type B in Table 2. After combining SCWT scores and structural MRI indices of the six brain regions as the classifying feature, better classification indices were obtained (feature type C in Table 2), which suggests cognitive scores may be helpful for the differential diagnosis of BD.

For accurate prediction, it was an important condition to select suitable features and feature dimensions. According to the above-mentioned work, we found that the feature importance in an excellent model is not the same as it behaved in a terrible model. A feature that is not important to a terrible model (poor cross-validation score) may be very important to an excellent model. Using six features retrained six classifiers, the value of feature importance had a new transformation. In this study, this phenomenon was especially prominent for the NB classifier. In Figure 3D, the importance of feature Pallidum.R was in fourth place, and its importance rose to first place in Figure 4D; the importance of feature MTG.R went up even more sharply. In Figure 4, the importance of the features was reordered, but the importance of the features MTG.R, Pallidum.R, and Pallidum.L had not changed and became more prominent. For classifier LR, SVM, NB, RF, kNN, and AdaBoost, the top three important features contain more than two of MTG.R, Pallidum.R, and Pallidum.L (Figure 4). It suggests that cortical thickness or volume of the three brain regions were very important features in the classification of PBD-I and HCs. On the other hand, the feature selection process of this study was implemented for the cross-validation process, not before the estimation of the ML model. It may avoid information leakage and excessive fitting to data samples (Claude et al., 2020).

Interestingly, all six classifiers showed the three features of MTG.R, Pallidum.R, and Pallidum.L, which is very important for the distinction between PBD-I and HCs. This result is in line with the abnormality of these three brain regions in PBD patients found in previous studies. It has been reported that the MTG may be involved in cognitive processes acting as a regulatory center for auditory information, such as language and semantic memory processing (Tranel et al., 1997; Chao et al., 1999; Cabeza and Nyberg, 2000). It has been proposed that MTG was associated with reduced cortical thickness in BD-I from an MRI analysis of 6,503 individuals (Hibar et al., 2018). Decreased MTG activation was found in patients with BD-I during the task of emotional images (Cerullo et al., 2014), which may reflect altered cognitive functions. Notably, abnormalities in MTG may serve as innovative biomarkers in BD for diagnosis and treatment. There is increasing evidence suggesting pallidal abnormalities in BD. An animal study demonstrated that the pallidum serves as a significant component of the cortico-striatal-pallido-thalamo-cortical (CSPTC) circuit involved in perception, attention, and emotion at downstream cortical levels (Galineau et al., 2017). It was also found that patients with severe mania had less intense activity in the right external segment of globus pallidus than HCs, suggesting that disorders in the right hemisphere may raise the manic signs (Caligiuri et al., 2003). Structural neuroimaging researchers have found smaller pallidal volume in BD patients (Abramovic et al., 2016) and in BD-I patients with a history of childhood trauma (Janiri et al., 2017). Additionally, functional neuroimaging findings have also demonstrated greater pallidual activity during a motor task in bipolar mania patients (Marchand and Yurgelun-Todd, 2010), and higher BOLD responses of left pallidum in BD mania groups (Caligiuri et al., 2003). The above-mentioned studies have indicated that there are structural impairments and functional dysfunction in the pallidum that perhaps represent more potential risk factors unique to the pathophysiology of BD.

## Limitations

The results of our work should be interpreted in a certain context, in particular the relatively small sample space. We realized that this aspect might be a limitation of our research, and we actually took measures, such as using *k*-fold cross-validation repeated *p* times. Moreover, sMRI was mainly used in this research, so the information contained was mainly anatomical structure information, which may not cover comprehensive enough information containing the cause of BD. In future research, our feature set would further add fMRI signals, such as magnetic resonance spectroscopy imaging (MRSI), and clinical information, such as various evaluation scales.

## Conclusion

The purpose of our study was to evaluate whether several ML algorithms could support distinguishing PBD-I and HCs. To this end, we implemented six different classifiers using the following algorithms: LR, SVM, RF, NB, kNN, and AdaBoost. The features were derived from 86 brain region MRI structural indices. For distinguishing PBD-I from HCs, eight features were selected by Lasso or f_classif. The six classifiers had different accuracy to classify PBD-I and HCs. The accuracy of LR, SVM, RF, and NB exceeded 80%, and RF had the highest accuracy of 84.53%, whereas the accuracy of kNN and AdaBoost was more than 75%. Meanwhile, it is worth noting that right MTG and bilateral pallidum play key roles in differentiating PBD-I and HCs, which is consistent with structural and functional anomalous changes in these brain regions in PBD patients.

## Data availability statement

The original contributions presented in this study are included in the article/Supplementary material, further inquiries can be directed to the corresponding author.

## Ethics statement

The studies involving human participants were reviewed and approved by the Ethics Committee of the Second Xiangya Hospital of Central South University. Written informed consent to participate in this study was provided by the participants or their legal guardian/next of kin.

## Author contributions

QJ and GL were involved in the conception and research design. WG and LS collected the clinical data. DC, JN, LK, and XZ processed the MRI data. RD performed the statistical analysis and wrote the manuscript. QJ, WC, YG, and JQ revised it for publication. All authors contributed to the article and approved the submitted version.

## Footnotes

1
https://surfer.nmr.mgh.harvard.edu

## Supplementary material

Supplementary Figure 1 Euler number of the PBD-I and HC groups. No significant difference was found in the Euler number between the two groups (two-sample *T*-test, *T*-value = 0.039, *P* = 0.969). Boxplots show the lower quartile (25%), median, and upper quartile (75%). The upper and lower whiskers represent the minimum and maximum, respectively.

## References

[B1] Abramovic L. Boks M. P. Vreeker A. Bouter D. C. Kruiper C. Verkooijen S. (2016). The association of antipsychotic medication and lithium with brain measures in patients with bipolar disorder. *Eur. Neuropsychopharmacol.* 26 1741–1751. 10.1016/j.euroneuro.2016.09.371 27665062

[B2] Acikel C. Aydin Son Y. Celik C. Gul H. (2016). Evaluation of potential novel variations and their interactions related to bipolar disorders: Analysis of genome-wide association study data. *Neuropsychiatr. Dis. Treat.* 12 2997–3004. 10.2147/NDT.S112558 27920536 PMC5127431

[B3] Baggenstoss P. M. (2004). Class-specific classifier: Avoiding the curse of dimensionality. *IEEE Aerosp. Electron. Syst. Mag.* 19 37–52. 10.1109/MAES.2004.1263230

[B4] Besga A. Gonzalez I. Echeburua E. Savio A. Ayerdi B. Chyzhyk D. (2015). Discrimination between Alzheimer’s Disease and Late Onset Bipolar Disorder Using Multivariate Analysis. *Front. Aging Neurosci.* 7:231. 10.3389/fnagi.2015.00231 26696883 PMC4677464

[B5] Breiman L. (2001). Random forests. *Mach. Learn.* 45 5–32. 10.1023/A:1010933404324

[B6] Cabeza R. Nyberg L. (2000). Imaging cognition II: An empirical review of 275 PET and fMRI studies. *J. Cogn. Neurosci.* 12 1–47. 10.1162/08989290051137585 10769304

[B7] Caligiuri M. P. Brown G. G. Meloy M. J. Eberson S. C. Kindermann S. S. Frank L. R. (2003). An fMRI study of affective state and medication on cortical and subcortical brain regions during motor performance in bipolar disorder. *Psychiatry Res.* 123 171–182. 10.1016/s0925-4927(03)00075-1 12928105

[B8] Cao B. Luo Q. Fu Y. Du L. Qiu T. Yang X. (2018). Predicting individual responses to the electroconvulsive therapy with hippocampal subfield volumes in major depression disorder. *Sci. Rep.* 8:5434. 10.1038/s41598-018-23685-9 29615675 PMC5882798

[B9] Cerullo M. A. Eliassen J. C. Smith C. T. Fleck D. E. Nelson E. B. Strawn J. R. (2014). Bipolar I disorder and major depressive disorder show similar brain activation during depression. *Bipolar Disord.* 16 703–712. 10.1111/bdi.12225 24990479 PMC4213254

[B10] Chao L. L. Haxby J. V. Martin A. (1999). Attribute-based neural substrates in temporal cortex for perceiving and knowing about objects. *Nat. Neurosci.* 2 913–919. 10.1038/13217 10491613

[B11] Chuang L. C. Kuo P. H. (2017). Building a genetic risk model for bipolar disorder from genome-wide association data with random forest algorithm. *Sci. Rep.* 7:39943. 10.1038/srep39943 28045094 PMC5206749

[B12] Claude L. A. Houenou J. Duchesnay E. Favre P. (2020). Will machine learning applied to neuroimaging in bipolar disorder help the clinician? A critical review and methodological suggestions. *Bipolar Disord.* 22 334–355. 10.1111/bdi.12895 32108409

[B13] Cortes C. Vapnik V. N. (1995). Support vector networks. *Mach. Learn.* 20 273–297. 10.1007/BF00994018

[B14] Doan N. T. Kaufmann T. Bettella F. Jorgensen K. N. Brandt C. L. Moberget T. (2017). Distinct multivariate brain morphological patterns and their added predictive value with cognitive and polygenic risk scores in mental disorders. *Neuroimage Clin.* 15 719–731. 10.1016/j.nicl.2017.06.014 28702349 PMC5491456

[B15] Domingos P. Pazzani M. (1997). On the Optimality of the Simple Bayesian Classifier under Zero-One Loss. *Mach. Learn.* 29 103–130. 10.1023/A:1007413511361

[B16] Freund Y. Schapire R. E. (1997). A Decision-Theoretic Generalization of on-Line Learning and an Application to Boosting. *J. Comput. Syst. Sci.* 55 119–139. 10.1006/jcss.1997.1504

[B17] Frias A. Palma C. Farriols N. (2015). Comorbidity in pediatric bipolar disorder: Prevalence, clinical impact, etiology and treatment. *J. Affect. Disord.* 174 378–389. 10.1016/j.jad.2014.12.008 25545605

[B18] Galineau L. Kas A. Worbe Y. Chaigneau M. Herard A. S. Guillermier M. (2017). Cortical areas involved in behavioral expression of external pallidum dysfunctions: A PET imaging study in non-human primates. *Neuroimage* 146 1025–1037. 10.1016/j.neuroimage.2016.10.039 27989846

[B19] Guyon I. Elisseeff A. (2003). An Introduction to Variable and Feature Selection. *J. Mach. Learn. Res.* 3 1157–1182. 10.1063/1.106515

[B20] Hibar D. P. Westlye L. T. Doan N. T. Jahanshad N. Cheung J. W. Ching C. R. K. (2018). Cortical abnormalities in bipolar disorder: An MRI analysis of 6503 individuals from the ENIGMA Bipolar Disorder Working Group. *Mol. Psychiatry* 23 932–942. 10.1038/mp.2017.73 28461699 PMC5668195

[B21] Janiri D. Sani G. Rossi P. Piras F. Iorio M. Banaj N. (2017). Amygdala and hippocampus volumes are differently affected by childhood trauma in patients with bipolar disorders and healthy controls. *Bipolar Disord.* 19 353–362. 10.1111/bdi.12516 28699182

[B22] Kuang L. Cui D. Jiao Q. Guo Y. Cao W. Gao W. (2020). Alterations of Cognition and Cerebral Ventricle Volume in Manic and Euthymic Pediatric Bipolar Disorder. *Front. Psychiatry* 11:593629. 10.3389/fpsyt.2020.593629 33381058 PMC7767823

[B23] Lemm S. Blankertz B. Dickhaus T. Muller K. R. (2011). Introduction to machine learning for brain imaging. *Neuroimage* 56 387–399. 10.1016/j.neuroimage.2010.11.004 21172442

[B24] Lin K. Shao R. Geng X. Chen K. Lu R. Gao Y. (2018). Illness, at-risk and resilience neural markers of early-stage bipolar disorder. *J. Affect. Disord.* 238 16–23. 10.1016/j.jad.2018.05.017 29852342

[B25] Marchand W. R. Yurgelun-Todd D. (2010). Striatal structure and function in mood disorders: A comprehensive review. *Bipolar Disord.* 12 764–785. 10.1111/j.1399-5618.2010.00874.x 21176024

[B26] Matsuo K. Harada K. Fujita Y. Okamoto Y. Ota M. Narita H. (2019). Distinctive Neuroanatomical Substrates for Depression in Bipolar Disorder versus Major Depressive Disorder. *Cereb. Cortex* 29 202–214. 10.1093/cercor/bhx319 29202177 PMC6294408

[B27] Mwangi B. Spiker D. Zunta-Soares G. B. Soares J. C. (2014). Prediction of pediatric bipolar disorder using neuroanatomical signatures of the amygdala. *Bipolar Disord.* 16 713–721. 10.1111/bdi.12222 24917530 PMC4234406

[B28] Nunes A. Schnack H. G. Ching C. R. K. Agartz I. Akudjedu T. N. Alda M. (2020). Using structural MRI to identify bipolar disorders - 13 site machine learning study in 3020 individuals from the ENIGMA Bipolar Disorders Working Group. *Mol. Psychiatry* 25 2130–2143. 10.1038/s41380-018-0228-9 30171211 PMC7473838

[B29] Peterson L. E. (2009). K-nearest neighbor. *Scholarpedia* 4:1883. 10.4249/scholarpedia.1883

[B30] Pirooznia M. Seifuddin F. Judy J. Mahon P. B. Bipolar Genome Study C. (2012). Data mining approaches for genome-wide association of mood disorders. *Psychiatr. Genet.* 22 55–61. 10.1097/YPG.0b013e32834dc40d 22081063 PMC3306768

[B31] Quintero Y. Ardila D. Camargo E. Rivas F. Aguilar J. (2021). Machine learning models for the prediction of the SEIRD variables for the COVID-19 pandemic based on a deep dependence analysis of variables. *Comput. Biol. Med.* 134:104500. 10.1016/j.compbiomed.2021.104500 34052570 PMC8142792

[B32] Rocha-Rego V. Jogia J. Marquand A. F. Mourao-Miranda J. Simmons A. Frangou S. (2014). Examination of the predictive value of structural magnetic resonance scans in bipolar disorder: A pattern classification approach. *Psychol. Med.* 44 519–532. 10.1017/S0033291713001013 23734914 PMC3880067

[B33] Rosen A. F. G. Roalf D. R. Ruparel K. Blake J. Seelaus K. Villa L. P. (2018). Quantitative assessment of structural image quality. *Neuroimage* 169 407–418. 10.1016/j.neuroimage.2017.12.059 29278774 PMC5856621

[B34] Salvador R. Radua J. Canales-Rodriguez E. J. Solanes A. Sarro S. Goikolea J. M. (2017). Evaluation of machine learning algorithms and structural features for optimal MRI-based diagnostic prediction in psychosis. *PLoS One* 12:e0175683. 10.1371/journal.pone.0175683 28426817 PMC5398548

[B35] Schnack H. G. Nieuwenhuis M. van Haren N. E. M. Abramovic L. Scheewe T. W. Brouwer R. M. (2014). Can structural MRI aid in clinical classification? A machine learning study in two independent samples of patients with schizophrenia, bipolar disorder and healthy subjects. *NeuroImage* 84 299–306. 10.1016/j.neuroimage.2013.08.053 24004694

[B36] Serpa M. H. Ou Y. Schaufelberger M. S. Doshi J. Ferreira L. K. Machado-Vieira R. (2014). Neuroanatomical classification in a population-based sample of psychotic major depression and bipolar I disorder with 1 year of diagnostic stability. *Biomed. Res. Int.* 2014:706157. 10.1155/2014/706157 24575411 PMC3915628

[B37] Struyf J. Dobrin S. Page D. (2008). Combining gene expression, demographic and clinical data in modeling disease: A case study of bipolar disorder and schizophrenia. *BMC Genom.* 9:531. 10.1186/1471-2164-9-531 18992130 PMC2628394

[B38] Tibshirani R. (2011). Regression shrinkage and selection via the lasso: A retrospective. *J. R. Statist. Soc. B* 73 273–282. 10.1111/j.1467-9868.2011.00771.x

[B39] Tranel D. Logan C. G. Frank R. J. Damasio A. R. (1997). Explaining category-related effects in the retrieval of conceptual and lexical knowledge for concrete entities: Operationalization and analysis of factors. *Neuropsychologia* 35 1329–1339. 10.1016/s0028-3932(97)00086-9 9347479

[B40] Zhang K. Hawkins C. Zhang Z. (2021). General-Purpose Bayesian Tensor Learning With Automatic Rank Determination and Uncertainty Quantification. *Front. Artif. Intell.* 4:668353. 10.3389/frai.2021.668353 35072057 PMC8777296

[B41] Zheng K. Wang X. (2017). Feature selection method with joint maximal information entropy between features and class. *Pattern Recognit.* 77 20–29. 10.1016/j.patcog.2017.12.008

