# Supplementary figures and images for: Machine learning algorithm performance evaluation in structural magnetic resonance imaging-based classification of pediatric bipolar disorders type I patients
Source: Front Comput Neurosci. 2022 Aug 23;16:915477. doi: 10.3389/fncom.2022.915477 (PMC9445985; doi:10.3389/fncom.2022.915477)

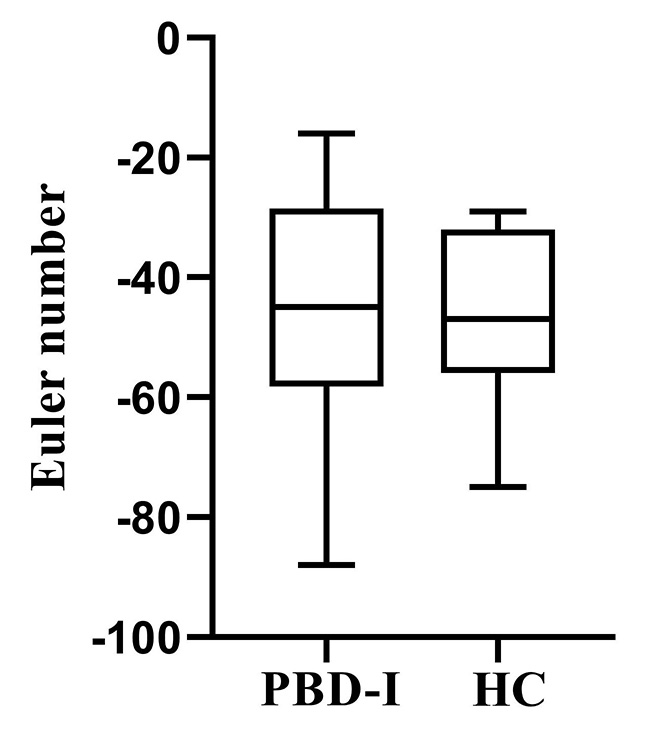

Supplement: Supplementary Figure 1 — Euler number of the PBD-I and HC groups. No significant difference was found in the Euler number between the two groups (two-sample T-test, T-value = 0.039, P = 0.969). Boxplots show the lower quartile (25%), median, and upper quartile (75%). The upper and lower whiskers represent the minimum and maximum, respectively. [file Image_1.jpeg]
